# Naturalistic use of psychedelics is associated with longitudinal improvements in anxiety and depression during global crisis times

**DOI:** 10.1177/02698811251346729

**Published:** 2025-06-18

**Authors:** Maria Bălăeţ, William Trender, Annalaura Lerede, Peter J Hellyer, Adam Hampshire

**Affiliations:** 1 Department of Brain Sciences, Imperial College London, London, UK; 2 Centre for Neuroimaging Sciences, Institute of Psychiatry, Psychology and Neuroscience, King’s College London, London, UK

**Keywords:** COVID-19, mental health, psychedelics, cannabis, depression, anxiety

## Abstract

**Background:**

Mental health implications of COVID-19 drug use patterns are still unclear.

**Methods:**

We used data-driven clustering in a large citizen science cohort recruited agnostically to an interest in drug-use to categorise people according to common patterns of drug use and analysed their mental health symptoms (GAD-7 and PHQ-9 items), from recruitment prior to COVID-19 restrictions in 2020 (*N* = 242,260) to three follow-ups in 2020-2022 (*N* = 68,416). Mixed effects modelling examined how mental health scores related to drug-use clusters cross-sectionally and how changes in those scores longitudinally related to changes in consumption frequencies.

**Results:**

We identified six common patterns of drug use during the COVID-19 pandemic, with cannabis cross cutting most of them. The majority of drug use clusters had worse average mental health scores relative to drug-naive individuals at all timepoints. The average mental health scores of those who used more drugs during the pandemic worsened over time relative to individual baselines. However, psychedelics and cannabis users showed average improvements in depression (β = −0.26 SD, 95% CI: −0.44, −0.08, *p* = 0.003), anxiety (β = −0.24 SD, 95% CI: −0.41, −0.06, *p* = 0.007) and overall mental health (β = −0.2 SD, 95% CI: −0.35, −0.04, *p* = 0.01) from pre-pandemic to January 2022, becoming on par with the drug-naive group. This was not the case for cannabis-only users, whose worse mental health scores persisted.

**Conclusion:**

Those who used psychedelics may have experienced some improvements in mental health across the pandemic timeframe, which supports the idea that beneficial effects on mood and anxiety associated with these substances may extend beyond controlled conditions.

## Introduction

The COVID-19 pandemic significantly disrupted daily life, profoundly affecting mental health (Hampshire et al., 2021a). Although overall rates of illicit drug use during the pandemic reduced, concurrently, there was a rise in their use as a coping mechanism (Bălăeţ et al., 2025; Rogers et al., 2020). However, longitudinal studies on the mental health of people who used illicit drugs spanning from before to after the pandemic restrictions are limited. In the UK, the need for such research is underscored by statistics indicating that 1 in 11 individuals aged 16–59 used drugs other than alcohol or tobacco in the past year (Office for National Statistics, 2023).

Of the few studies that specifically examined the relationship of substance use with mental health during the pandemic, most have focused on alcohol, tobacco or cannabis, often grouping other psychoactive substances and overlooking their unique effects (Zolopa et al., 2022). However, recent efforts have targeted the psychedelics and entactogens associations with mental health outcomes such as mood and resilience, distinct from other drugs (Kopra et al., 2023). This is timely given the increased interest in the potential use of psychedelics for therapeutic purposes (Carhart-Harris et al., 2021) and their use among the general population to try and self-manage well-being, including those without severe mental health issues (Keyes and Patrick, 2023; Livne et al., 2022). This interest is driven by the therapeutic potential of psychedelics like psilocybin for severe depression (Carhart-Harris et al., 2021), as well as research suggesting mood-enhancing effects in ‘naturalistic settings’, that is, as they are commonly used in the real world (Forstmann et al., 2020).

Research on the relationship between psychedelics use during the COVID-19 pandemic and mental health has produced mixed findings. Some studies, like those by Livne et al. (2022) and Keyes and Patrick (2023), cautioned against potential harms from increasing use, but have received criticisms based on reports of positive associations between psychedelics use during the pandemic with well-being (Morgan, 2023). The evidence for these positive associations comes from social media-based studies (Bouso et al., 2023; Cavanna et al., 2021; Révész et al., 2021), but contrasts with findings from large-scale surveys in the United Kingdom and the United States (Bălăeț et al., 2023b; Matzopoulos et al., 2021). These larger surveys indicate negative mental health correlations with psychedelics but a positive association for 3,4-methylenedioxymethamphetamine (MDMA; Bălăeț et al., 2023b), suggesting that assessing MDMA and psychedelics together, often employed by historic surveys, may contribute to inconsistent results. Discrepancies might also stem from recruitment bias, methodological differences and cultural factors. This highlights a need for more detailed longitudinal analyses to better understand mental health dynamics among psychedelic users, non-users, and users of other illicit drugs in the context of real-world stressors like the pandemic.

Studying drug use in the general population presents several challenges, particularly due to the prevalence of polydrug use; specifically, individuals often use multiple substances across time and may consume them simultaneously (Bălăeț et al., 2023b; de Jonge et al., 2022). This complicates efforts to isolate and model the effects of individual drugs, as it diverges from common patterns of drug use in the general population. Furthermore, drug use behaviour is dynamic, changing over time, adding another layer of complexity. Addressing these issues requires large-scale population sampling, which gathers data from real-world drug users in the context of environmental stressors and accounts for the varied and evolving drug use patterns observed in everyday life.

Here, we aim to advance our understanding of the relationship between mental health symptoms and common patterns of illicit drug use in the UK general population during crisis times. Specifically, we analyse online survey data collected from a large citizen science cohort spanning the timeframe from just prior to COVID-19 restrictions being put in place in the UK until early 2022. We test the hypothesis that those who use psychedelics had less frequent depression and anxiety symptoms across timeframe relative to their baselines, and relative to those who used other illicit drugs or who were drug naive.

## Methods

### Participant’s recruitment

Individuals were enrolled through the Great British Intelligence Test (Hampshire, 2020) via two distinct phases, with outreach conducted via the British Broadcasting Corporation (BBC)’s online platform and a BBC2 Horizon documentary. Initial enrolment spanned from December 2019 to January 2020, followed by a subsequent phase in May 2020; however, the portal for enrolment remained accessible continuously during this interval. Those who opted to share their email addresses at the time of enrolment were approached for follow-up in December 2020 and again in June 2021 and January 2022.

The main purpose of the Great British Intelligence Test was to understand human cognition and how cognitive performance relates to various health and lifestyle factors. This study was hosted on the Cognitron cognitive testing platform (Bălăeţ et al., 2024; Del Giovane et al., 2023; Hampshire et al., 2021b). The participants were informed that the cognitive testing battery would be paired with a questionnaire surveying health and lifestyle factors. However, no direct mention of the survey containing questions about drug use was made in the recruitment advertisement materials, thus minimising the potential bias of participants volunteering to take part in drug-focused research.

At recruitment (in 2019–2020) *N* = 95,441 participants made accounts on the Cognitron platform and agreed to be re-contacted for future research participation, with this number growing continuously to *N* = 124,496 by January 2022. Up to one in five of those who consented to be recontacted engaged with at least a single follow-up. No attempt has been made to mitigate the lack of responses from other members of the cohort.

This study was run in accordance with the Helsinki Declaration of 1975, as revised in 2008. All procedures were approved by the Imperial College Research Ethics Committee (17IC4009). All participants provided informed consent prior to completing the survey.

### Mood self-assessment

Selected items from the PHQ-9 (Kroenke and Spitzer, 2002) and the GAD-7 (Spitzer et al., 2006) were included in each of the analysis timepoints. To reflect mood fluctuations more comprehensively, we requested participants to reflect on their mood over the past month leading up to the survey, extending beyond the original 2-week timeframe of the standard scales. To gain a finer resolution of mood variations, we expanded the scoring scale to capture a broader spectrum of frequency (Bălăeț et al., 2023a, 2023b; Hampshire et al., 2021a). Participants rated the occurrence of symptoms over the previous month on a scale ranging from 0 to 6, with the points defined as follows: ‘0-Never’, ‘1-Almost never’, ‘2-Once or twice a week’, ‘3-Several times a week’, ‘4-Daily’, ‘5-Hourly’ and ‘6-More often’.

These are the items:

1. Feeling nervous, anxious or on edge – from GAD-7.
2. Feeling down or depressed – from PHQ.
3. Feeling tired or having little energy – from PHQ.
4. Trouble concentrating on things, such as reading the newspaper or watching television – from PHQ.
5. Not being able to get to sleep or stay asleep – from PHQ.

### Drug use assessment

During the months of December 2020, June 2021 and January 2022, participants had the opportunity to provide information regarding their use of drugs classified as illegal within the UK. Depending on their willingness to engage with this aspect of the survey, respondents were categorised into groups:

A. Undisclosed: This group includes those who chose not to reveal their past drug use and were not included in subsequent analyses.
B. Drug naive: Participants who indicated that they have never taken recreational drugs other than alcohol and tobacco.
C. Users: This group consists of individuals who acknowledged the use of illicit drugs beyond alcohol and tobacco at some point in their lifetime (specifically, we surveyed use of cannabis, cocaine, heroin/opioids, ayahuasca, psilocybin (magic mushrooms), MDMA/Ecstasy, lysergic acid diethylamide (LSD), 5-methoxy-N,N-dimethyltryptamine, mescaline, N,N-dimethyltryptamine, ketamine and ‘other’). Those who indicated using drugs at some point in their lifetime, but not in 2019 or during the pandemic, were classed as historic users. Those who used drugs up until the pandemic but not during the pandemic were classed as users who stopped using drugs during the pandemic. Further subdivision within this category was executed based on their specific choices of drugs used during the pandemic (2020–2022), utilising k-modes clustering to delineate specific clusters (Supplemental Figure S2).

### K-modes clustering

K-modes clustering was utilised to classify individuals based on their self-reported drug use, offering a data-driven method tailored for categorical data. Each individual is represented by a set of *N* features, encapsulating the types of drugs used during the pandemic and their historical usage patterns. The algorithm assigns each individual to the cluster whose mode is most similar to them, based on a predefined distance metric. In our analysis, the individual drug user serves as the data point, and the features include various drug class choices used in the year prior to the cognitive assessment, coded in binary terms.

A five-fold cross-validation method was used on an 80% train, 20% test split (Gholamy et al., 2018) of the drug survey responses across the three follow-ups to identify the ideal number of clusters. The optimal number for the data was identified as six clusters (see Supplemental Materials Figures S1 and S2), and the trained model was used to assign cluster labels to all data points. The clusters were then used as groups in the statistical analysis.

### Statistical assessment

A factor analysis with one factor was used to define the mental health composite score. This was done with the factor-analyser package in Python (Biggs, 2019).

All mental health self-assessment scores were adjusted for the effects of relevant covariates (sociodemographic characteristics – age decade, sex, ethnicity, residence, occupation) and lifestyle choices (reading, meditation and exercise frequency) using linear regression models. Residuals from these models are used for all further analysis. This is consistent with data analysed in previous publications (Bălăeţ et al., 2023b, 2025).

Mixed effects linear models with cluster as a between-subjects factor and time as a within-subjects factor were used to assess each set of changes in mental health symptom scores from baseline to follow-up independently.

### Permutation analysis description

Two-way permutational ANOVA was applied to evaluate the effects of cluster, changes in drug use and their interaction on changes in all mental health scores. Changes in drug use and individual scores were evaluated between each follow-up and baseline. To avoid permuting individuals with themselves at another timepoint, changes in mental health scores were permuted across individuals with the same number of follow-up timepoints available. For example, both observations from individuals with two follow-up time points available were permuted only with individuals with two follow-up timepoints available. This approach was similar to what was applied in Lerede et al. (2023) and enabled controlling for the level of engagement as a potential confounder as well as respecting the hypothesis of independence among observations to permute.

All analyses were performed using the Python package ‘statsmodel’ (Seabold and Perktold, 2010).

## Results

### Study timeline and participation

**Figure 1.**
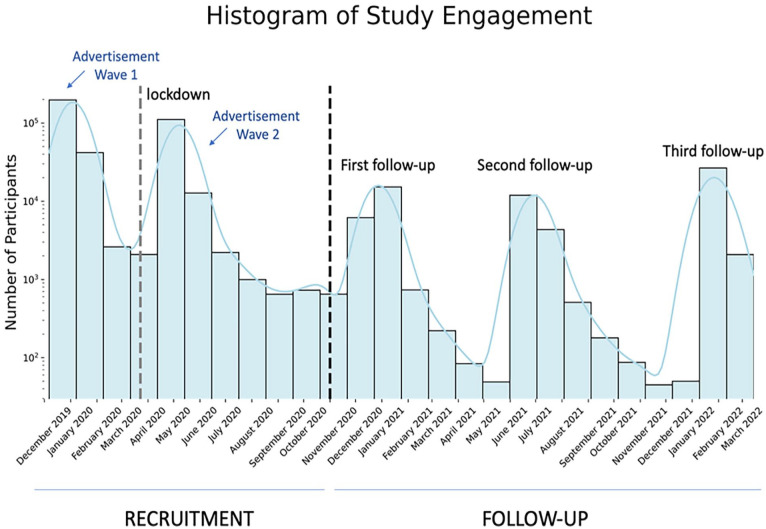
Study engagement timeline. The log scale histogram represents the number of individuals who completed our survey between late December 2019 and March 2022. The study website was made widely visible by the BBC at two time points from January to February 2020 and May 2020. On 23 March 2020, a lockdown due to the COVID-19 pandemic began in the UK. Participants continued to engage with the recruitment website in the absence of advertisement until the end of 2020, prior to our first follow-up of consenting individuals.

Overall, the study engaged *N* = 377,678 unique individuals between December 2019 and March 2022, spanning five distinct phases: two initial advertisement waves (December 2019–May 2020) and three subsequent recontact waves at 6-monthly timepoints following recruitment (December 2020, June 2021, January 2022; (Figure 1). We recruited a total of *N* = 242,260 datasets pre-COVID-19 restrictions and *N* = 130,505 during the first months of restrictions initially being put in place. Subsequently, we collected *N* = 68,416 datasets at follow-ups across three timepoints: *N* = 22,533 datasets in December 2020, *N* = 17,172 in June 2021 and *N* = 28,711 in January 2022.

## Participants clustering

The K-modes clustering algorithm was used to cluster all recontacted individuals based on their reported patterns of drug use during the COVID-19 pandemic. One participant could only belong to one cluster. Six distinct clusters were identified comprising those who used: cannabis (*N* = 2827), cocaine and cannabis (*N* = 393), cocaine only (*N* = 629), psychedelics and cannabis (*N* = 430), polydrug (*N* = 274) and those indicating ‘other’ (*N* = 283; Supplemental Figure S2). Users of ‘other’ drugs were excluded from subsequent analyses due to the inability to pinpoint what the substances were. The remaining datasets belonged to *N* = 32,491 drug-naive participants, *N* = 10,293 with a history of drug use and *N* = 1420 who stopped using drugs during the pandemic.

Here, only complete datasets from participants recruited prior to restrictions in the UK and who responded to at least one recontact timepoint (thus completing another set of mental health measures alongside indicating whether they have used drugs during the COVID-19 pandemic) were used in our analyses. A total of *N* = 48,757 follow-up entries were retained, belonging to 30,711 unique individuals who completed at least one recontact. Among them, *N* = 17,354 participants engaged in just one recontact, while *N* = 8668 and 4689 individuals completed two and three recontacts, respectively. These included *N* = 15,591 drug-naive individuals, *N* = 5738 historic users, *N* = 857 individuals who stopped using drugs during the pandemic, *N* = 1515 cannabis users, *N* = 306 cocaine users, *N* = 228 psychedelics and cannabis users, *N* = 210 cocaine and cannabis users and *N* = 136 polydrug users. Sociodemographic characteristics and lifestyle choices of the participants who entered the longitudinal mental health analysis are reported in Supplemental Table S1, whereas the cohort sociodemographics at recruitment and cross-sectionally at follow-up have been reported in previous publications (Bălăeț et al., 2023a, 2023b; Hampshire et al., 2021a).

## The bigger picture: Longitudinal changes in mental health for different clusters

**Figure 2.**
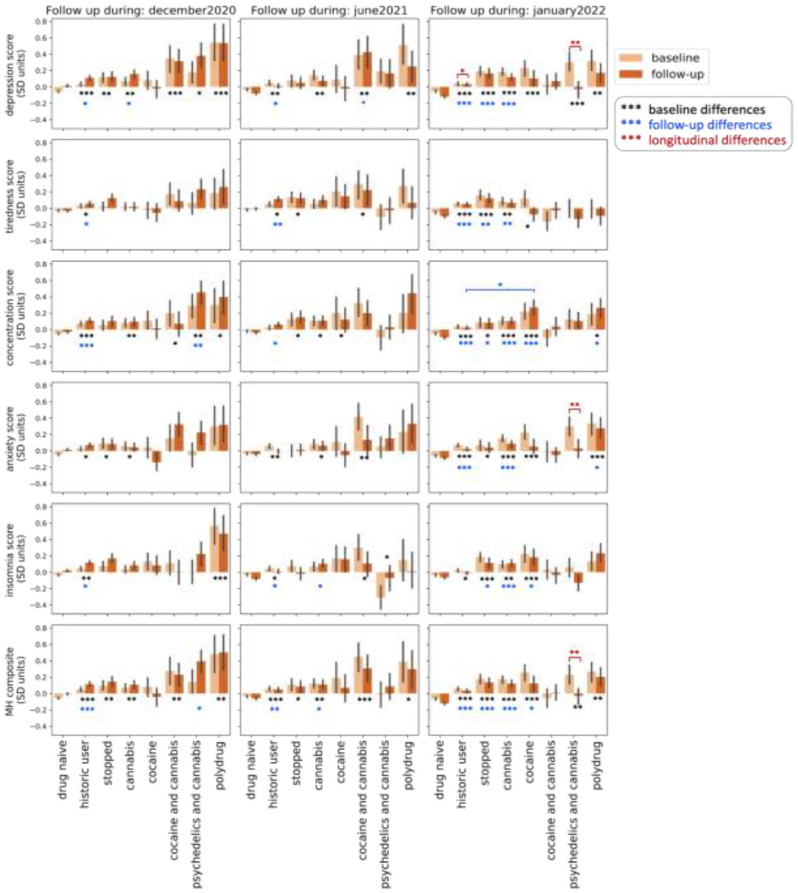
Changes in mood self-assessment scores from pre-COVID-19 restrictions to follow-up. Error bars are the standard error of the mean. A full breakdown of numbers per cluster at each recontact is available in Supplemental Table S2. Significance for cluster effects is annotated based on cluster predictors in the mixed effects linear model. The significance annotation for cluster effects is black and represents whether there were baseline differences between clusters relative to drug-naïve individuals. Significance for changes over time is annotated based on the significant interactions between time and cluster from the mixed effects linear model results. The significance annotation for interactions is in red and represents whether there have been significant longitudinal changes over time from baseline to follow-up. The significance annotation for group differences at follow-up is based on post hoc Tukey tests where ANOVA indicated an effect of cluster and is represented in blue. This represents differences relative to drug-naïve individuals unless otherwise indicated on the figure. Baseline and follow-up differences are between clusters. Longitudinal differences are within clusters. All *p*-values are annotated as follows: *p < 0.05. **p < 0.01. ***p < 0.001.

Full mixed effects model outputs are reported in the supplement (Supplemental File 1). In summary, at baseline, drug use clusters had generally worse mental health scores relative to drug-naive individuals for all mental health symptoms and across all timepoints. This included historic users and those who stopped using drugs during the pandemic (Figure 2).

Regarding the psychedelics and cannabis cluster specifically, there was an effect of cluster indicative of worse baseline depression scores (β = 0.23 SD, 95% CI: 0.003, 0.46, *p* = 0.04) and worse baseline concentration scores (β = 0.34 SD, 95% CI: 0.10, 0.57, *p* = 0.003) for those who responded in December 2020 relative to drug-naive individuals. Psychedelics and cannabis users who responded in June 2021 had better baseline insomnia scores (β = −0.28 SD, 95% CI: −0.56, −0.004, *p* = 0.04) than drug-naive individuals, while psychedelics and cannabis users who responded in January 2022 had worse depression scores (β = 0.35 SD, 95% CI: 0.16, 0.54, *p* < 0.001) and worse mental health composite scores (β = 0.29 SD, 95% CI: 0.10, 0.48, *p* = 0.002) than drug-naive individuals. Cannabis-only users, irrespective of the timepoint they engaged with a follow-up, had on average worse baseline depression, concentration, anxiety and mental health composite scores relative to drug-naïve individuals. All average mental health scores of cannabis-only users who responded in January 2022 were worse at baseline relative to drug-naïve individuals.

For depression scores, there was a significant interaction for historic users (β = 0.06 SD, 95% CI: 0.011, 0.014, *p* = 0.01) who had worse depression scores in January 2022 and psychedelics and cannabis users who had better depression scores in January 2022 (β = −0.26 SD, 95% CI: −0.44, −0.08, *p* = 0.003). In January 2022, there was also a significant interaction for psychedelics and cannabis users who had better anxiety scores (β = −0.24 SD, 95% CI: −0.41, −0.06, *p* = 0.007). For the composite mental health score, in January 2022, there was a significant interaction between timepoint and the psychedelics and cannabis cluster (β = −0.2 SD, 95% CI: −0.35, −0.04, *p* = 0.01). This was not the case for those who only used cannabis during the same period of time, who presented with no longitudinal changes in any of the variables.

ANOVA and Tukey post hoc tests were carried out to investigate differences between clusters at follow-up. The main cluster of interest, psychedelics and cannabis, showed worse concentration scores (mean difference = 0.48 SD, 95% CI: 0.11, 0.85, *p* = 0.002) and worse composite mental health scores (mean difference = 0.39 SD, 95% CI: 0.02, 0.76, *p* = 0.02) relative to drug-naive individuals in December 2020. They were no different relative to drug-naive individuals in June 2021 or January 2022 in any mental health symptoms. However, in January 2022, cannabis-only users were significantly worse than drug-naive individuals for every mental health symptom. Full results are reported in Supplemental File 2.

## Changes in frequency of use across drug use clusters

At all timepoints, most individuals who used drugs during the pandemic reported no change in frequency of use from their pre-restriction levels. Approximately one in four individuals reported using drugs less frequently, and one in five reported using them more frequently. Distinct patterns were observed for different drug use clusters at different timepoints, which differed from one another. Just above half of psychedelics and cannabis users reported no change from their baseline levels, with the rest being almost evenly split between using less and using more, a pattern observed at all follow-up timepoints.

## Relating changes in mental health symptoms to changes in the frequency of drug use

**Figure 3.**
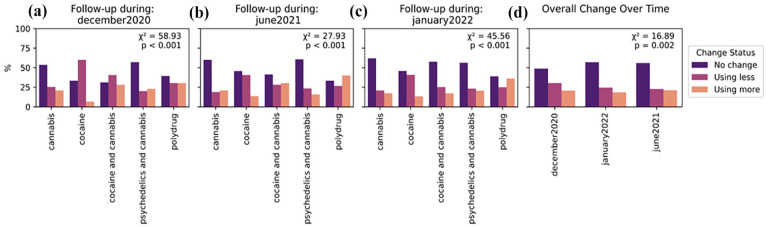
Self-reported change in drug use frequency from pre-restrictions to follow-up. Proportions of respondents who indicated using less drugs, more drugs or no change in their drug use frequency are represented for each of the clusters at follow-up (a–c) and overall (d).

Finally, we examined whether there were differences in the changes from pre-restrictions to follow-up in the different mental health symptoms deltas (difference in scores between pre-restrictions to follow-up) based on users’ self-reported change in frequency of drug use from pre-pandemic times to follow-up. Positive deltas indicate a worsening of scores, whereas negative deltas indicate an improvement in scores (Figure 3). Segregation by drug cluster is reported in Supplemental Materials.

**Figure 4.**
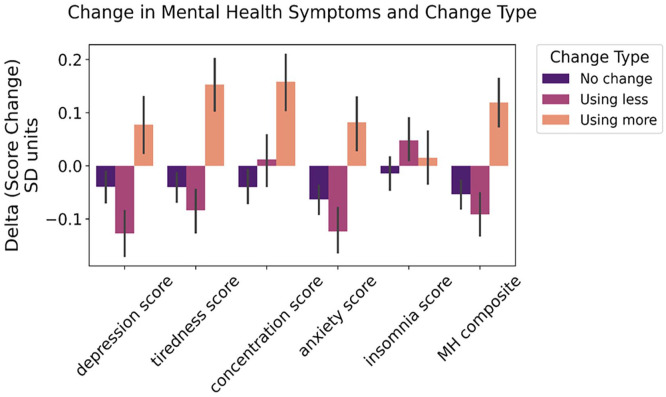
Differences in changes from pre-restrictions to post-restrictions based on changes in drug use. Error bars are the 95% confidence interval. A breakdown of numbers per cluster per change is available in Supplemental Materials Table S3.

Results from the two-way permutational ANOVA (Figure 4) showed that changes in composite mental health were associated with changes in drug use, regardless of cluster, evidenced by a significant main effect of change (*F* = 7.03, *p* = 0.001), but not of cluster or an interaction between change and cluster. For depression, there was a significant main effect of change (*F* = 4.78, *p* = 0.01), but not of cluster or interaction. For anxiety, there was a significant main effect of change (*F* = 3.54, *p* = 0.04), but the main effect of cluster and the interaction term were not significant. For concentration, the main effect of change was significant (*F* = 6.56, *p* = 0.001), but not of cluster or interaction. For tiredness, there was a significant main effect of change (*F* = 9.07, *p* < 0.001) and a significant interaction (*F* = 2.49, *p* = 0.01), but not of cluster. For insomnia, there were no significant main effects of change, cluster or interaction.

Supplemental Figure S4 illustrates the split per cluster.

## Zooming in on the psychedelics and cannabis cluster changes in drug use levels and mental health symptoms

**Figure 5.**
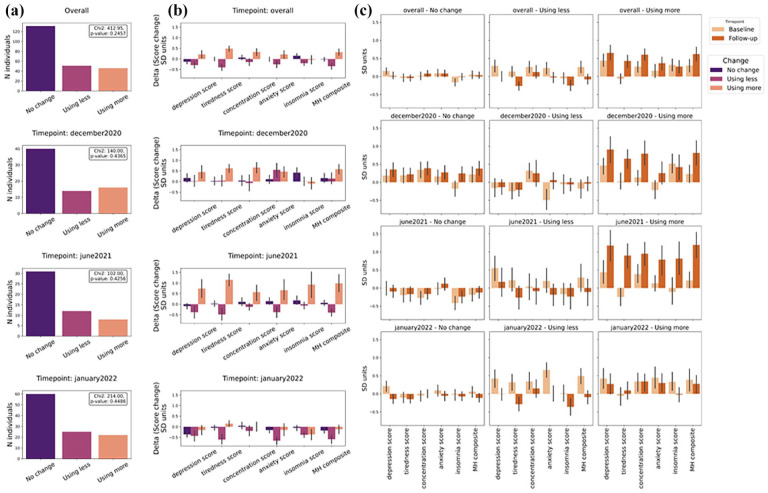
Changes in use and mental health scores of psychedelics and cannabis users. (a) Distribution of changes in use in the participants who used psychedelics and cannabis during the COVID-19 pandemic. (b) Mental health score by changes at each timepoint for those who used psychedelics and cannabis during the COVID-19 pandemic. (c) Changes from baseline to follow-up within different change groups for psychedelics and cannabis users. All error bars are standard errors of the mean.

There was no statistically significant difference in the number of participants who used psychedelics and cannabis during the COVID-19 pandemic and indicated a different type of change relative to their self-perceived baseline before the pandemic (Figure 5(a)). A two-way ANOVA analysis revealed a main effect of change in June 2021 (*F*(2, 288) = 20.54, *p* < 0.001) and a main effect of change in January 2022 (*F*(2, 624) = 6.72, *p* < 0.001) but no effect of mental health score, nor an interaction between the two at any timepoints. Those who used more by June 2021 had worse mental health scores, whereas those who used less by January 2022 had better scores at that time (Figure 5(b)). Longitudinally, those who used more drugs during the pandemic had worse mental health scores in December 2020 and June 2021 relative to their baseline scores, whereas those who used less had better scores in January 2022 relative to their baseline scores (Figure 5(c)).

## Discussion

This study examined how mental health evolved over time in relation to different patterns of illicit drug use during the COVID-19 pandemic within a large citizen science cohort. We identified six distinct clusters of drug users, with cannabis only emerging as the most frequent pattern, followed by cocaine, cocaine and cannabis, psychedelics and cannabis and polydrug use. These clusters are consistent with established naturalistic drug user behaviours (Hakkarainen et al., 2019) and have been replicated using different algorithms (Bălăeț et al., 2023b). They also match the official order of prevalence of substances being used in the UK during the period of time the study ran (Office for National Statistics, 2023). Together, these aspects underscore the ecological validity of using a data-driven clustering approach for studying associations between drug use and other variables.

Significant differences in mental health scores were observed before the pandemic between those who went on to use drugs during the pandemic and drug-naive individuals. These findings are consistent with the literature suggesting drug users have generally worse mental health than non-users (Torrens et al., 2011). We also found that those who increased their drug use frequency during the pandemic, irrespective of what drugs they used more of, had worse mental health scores during the pandemic. Previous research has shown a direct relationship between the quantity of drug use and more severe mental health issues (Ringen et al., 2008).

Mental health in most drug use clusters remained stable over time, except for the psychedelics and cannabis cluster. At follow-up, this cluster showed significant within-subject improvements. Comparing pre-restrictions data to January 2022, individuals in this group had significantly worse depression and mental health composite scores than drug-naive individuals at baseline, but these differences diminished over time, with no significant differences relative to drug-naive individuals remaining at follow-up. Anxiety scores also dropped significantly in this cluster, though the differences from drug-naive individuals did not reach statistical significance at either baseline or follow-up. Further analyses suggest this might be due to individuals in this cluster generally using fewer drugs by January 2022 relative to their pre-pandemic baseline. By contrast, cannabis-only users consistently showed poorer mental health across all symptoms compared to drug-naive individuals, suggesting the change in mental health scores might be related to the additional use of psychedelics within that cluster.

This observation accords with previous findings that link naturalistic use of psychedelics to improved mental health (Forstmann et al., 2020), and is consistent with Bouso et al. (2023), a longitudinal analysis carried out during the pandemic on psychedelics users. Improvements in mental health have also been reported in within-subject design studies of psychedelics users in naturalistic settings (Nygart et al., 2022). Our analysis also adds further nuance to the findings previously presented in Bălăeț et al. (2023b); specifically, in that study, we found that those who went on to use drugs during the peak of the COVID-19 pandemic, including those in the psychedelics and cannabis cluster, had worse mental health scores than drug-naive individuals during that time. Here, we illustrate that while cross-sectionally psychedelics and cannabis users indeed had worse mental health before and during the peak of the pandemic, in a within-subjects design, there were improvements, and an eventual normalisation of mental health scores to be on par with drug-naive individuals by 2022, when most restrictions were already lifted in the UK.

There are several pertinent explanations for this result. At the population level, drug users have worse mental health than drug-naive individuals – and it could be that (novel) use of psychedelics, albeit less drug use more generally, during crisis times, normalises those differences. Another possible explanation is that the context is more influential in driving the effects of psychedelics than it is in driving the effects of other drugs. We note that related to the early stages of the pandemic, when restrictions and uncertainty were more severe in the UK, the participants in the psychedelics and cannabis cluster who used more drugs had worse mental health scores at those timepoints relative to their pre-restrictions baselines. This contrasts with participants surveyed later on, when restrictions were more relaxed and a lot of things started returning to normal, in January 2022, who had similar mental health scores to their pre-restrictions baselines, despite using more drugs. This explanation is in line with previous work discussing increased suggestibility during the psychedelic experiences (Carhart-Harris et al., 2018), the importance of set and setting (Hartogsohn, 2017) and how the general societal discourse could affect users (Bălăeţ, 2024; Bunce, 1979). A potential mechanistic explanation for the improvements observed in the psychedelics and cannabis cluster may also relate to the longer interval between baseline and follow-up when these improvements were noted (approximately 2 years, compared to 1 or 1.5 years in the other analyses). This extended timeframe could imply either that psychedelic use occurred more proximally to the point of survey, coinciding with a relatively stable post-pandemic context conducive to more positive outcomes, or that sufficient time had elapsed since earlier use to allow for psychological integration, thereby supporting longer-term improvements in mental health.

Alternative explanations should also be considered. One possibility is regression to the mean – a statistical phenomenon in which extreme scores at one timepoint tend to move closer to the population average upon subsequent measurement. Given the relatively poorer mental health at baseline in the psychedelics and cannabis cluster, and the apparent normalisation of these measures at follow-up, it is plausible that some of the observed improvement reflects this artefact rather than a specific effect of psychedelic use. Another non-causal interpretation is that individuals whose mental health was already improving in the aftermath of the COVID-19 pandemic may have been more inclined to experiment with psychedelics nearer to the time of the final survey. However, as the timing of psychedelic use was not recorded, it is not possible to determine whether improvements in mental health preceded or followed psychedelic use. Additional explanations include selective engagement with research, whereby participants with less positive experiences may have been less likely to remain engaged with the study, or the influence of unmeasured third variables that independently contributed to improved outcomes.

As our data-driven clustering reveals, virtually all people who used psychedelics also used cannabis during the pandemic, making it impractical to study the association of naturalistic psychedelics use with mental health in isolation. Looking at the cannabis cluster during the pandemic’s peak revealed notably worse mental health scores compared to drug-naive individuals across multiple symptoms, including anxiety, concentration, insomnia and tiredness, though the average mental health of this group did not significantly change over time. The associations between cannabis use and mental health are widely debated – most notably, there is evidence that it can amplify anxiety and paranoia in users while it is also being clinically prescribed for treating anxiety (Van Ameringen et al., 2020). Furthermore, there is evidence to suggest that heavy cannabis use may increase the risk of developing depression (Lev-Ran et al., 2014). Conversely, experimental findings show conflictual evidence that cannabis users typically exhibit blunted reactivity to negative emotions (Somaini et al., 2012) and stress (Cuttler et al., 2017), which, in turn, might help them cope with such symptoms. Evidence suggests consuming cannabis at the same time as psychedelics may make psychedelic experiences more intense in a dose-dependent fashion (Kuc et al., 2022), which may explain potential negative associations between combined use and worse mental health. However, since we do not have information on whether the use of psychedelics and cannabis occurred simultaneously or asynchronously across our cohort, it is not possible to firmly draw such conclusions. Intense experiences may provide grounds for improved mental health with adequate integration (Gashi et al., 2021), or benefits from psychedelics use may be independent of cannabis use during the same timeframe. Given the prevalence of combined psychedelics and cannabis use, a timely challenge for future studies is to determine how these drugs interact with each other and their effects on mental health, which may only be achievable under prospective study designs with sufficient power to map out nuanced drug use patterns.

Observational studies of naturalistic drug use present a cost-effective opportunity for understanding the associations between drug use and mental health outcomes and could help inform studies aimed at developing these substances as therapies, which are otherwise logistically challenging to carry out (Bălăeţ, 2025; Barnett, 2025; Barnett et al., 2022, 2025). Strengths of our study include that we addressed the relationship between recreational drug use and mental health in unsupervised settings while minimising the selection bias common when recruiting in drug-related social media forums. This was achieved through a recruitment approach that did not mention drug-related questions and avoided promotion on drug-centric social media channels. The participant group was diverse, including drug-naive individuals, historic users, and those who stopped using drugs during the COVID-19 pandemic; although diversity in sociodemographic characteristics, expectedly, dwindled with repeated follow-up. The methodology was adapted to recognise the complexity of drug use behaviours, including the concurrent use of cannabis with other substances, and to reflect the dynamic nature of drug use patterns where participants increased or decreased their use. Our study also has limitations. As data collection was fully automated online, we did not conduct the sorts of interviews that are sometimes used to provide comprehensive baseline data on participants’ drug use histories, which limits our ability to assess the influence of prior drug use on mental health. For instance, we did not collect data pertaining to dosage, frequency or context of drug use, which are likely important in determining mental health outcomes, nor have we gathered specific information on other drugs individuals may use in the UK, such as amphetamines. We also did not control for potential confounding effects of psychiatric medications that participants might have taken. Furthermore, participants who engaged in the study at multiple timepoints may be less likely to represent all patterns of naturalistic drug use as some sub-populations, for example, those who have addictions or are otherwise marginalised, may be less likely to engage in research. The focus of the analysis was solely on illicit drugs (in the UK), omitting licit substances like alcohol and tobacco – those data will form the basis of future analyses. Finally, the sample predominantly comprised British participants, which may limit the generalisability of the findings.

Taken together, our findings demonstrate that, on average, individuals who use drugs report poorer mental health compared to drug-naïve individuals, and these average differences remained relatively stable from before the COVID-19 restrictions to the years following their implementation. Notably, the cluster comprising psychedelics and cannabis use exhibited distinct temporal changes, with average anxiety and depression scores improving from pre-restriction baselines to the final follow-up. Additionally, we observed that increases in drug use were associated with declines in mental health over time, regardless of the specific substances involved. It is important to recognise that these results reflect population-level trends and do not account for individual variations in mental health trajectories. Future research should investigate whether the observed changes in mental health within the psychedelics and cannabis cluster are driven by alterations in the use of cannabis, psychedelics or their combined effects, particularly given their prevalent concurrent use; or whether they are a product of other synergistic or independent factors (such as the quality of interpersonal relationships, concurrent treatment for mood disorders or lifestyle changes).

## Supplemental Material

sj-docx-1-jop-10.1177_02698811251346729 – Supplemental material for Naturalistic use of psychedelics is associated with longitudinal improvements in anxiety and depression during global crisis times Supplemental material, sj-docx-1-jop-10.1177_02698811251346729 for Naturalistic use of psychedelics is associated with longitudinal improvements in anxiety and depression during global crisis times by Maria Bălăeţ, William Trender, Annalaura Lerede, Peter J Hellyer and Adam Hampshire in Journal of Psychopharmacology

sj-txt-2-jop-10.1177_02698811251346729 – Supplemental material for Naturalistic use of psychedelics is associated with longitudinal improvements in anxiety and depression during global crisis times Supplemental material, sj-txt-2-jop-10.1177_02698811251346729 for Naturalistic use of psychedelics is associated with longitudinal improvements in anxiety and depression during global crisis times by Maria Bălăeţ, William Trender, Annalaura Lerede, Peter J Hellyer and Adam Hampshire in Journal of Psychopharmacology
