# Supplementary material for: Naturalistic use of psychedelics is associated with longitudinal improvements in anxiety and depression during global crisis times
Source: J Psychopharmacol. 2025 Jun 18;39(9):957–67. doi: 10.1177/02698811251346729 (PMC12371141; doi:10.1177/02698811251346729)
Supplement: sj-docx-1-jop-10.1177_02698811251346729 – Supplemental material for Naturalistic use of psychedelics is associated with longitudinal improvements in anxiety and depression during global crisis times [file sj-docx-1-jop-10.1177_02698811251346729.docx]

**Supplementary Materials**


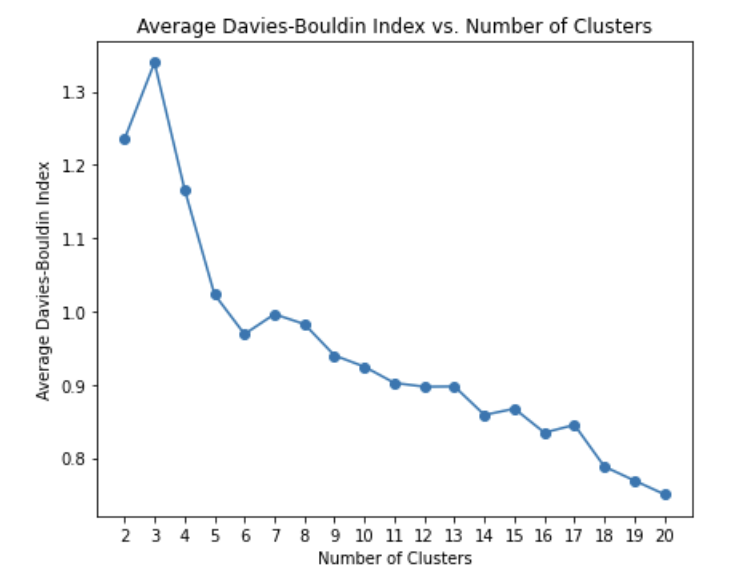


**Supplementary Figure 1. Davies-Bouldin Index for different cluster splits.** The Davies-Bouldin index was used to determine the optimal number of k-modes clusters. This was chosen as the first ‘dip’ of the lowest N.


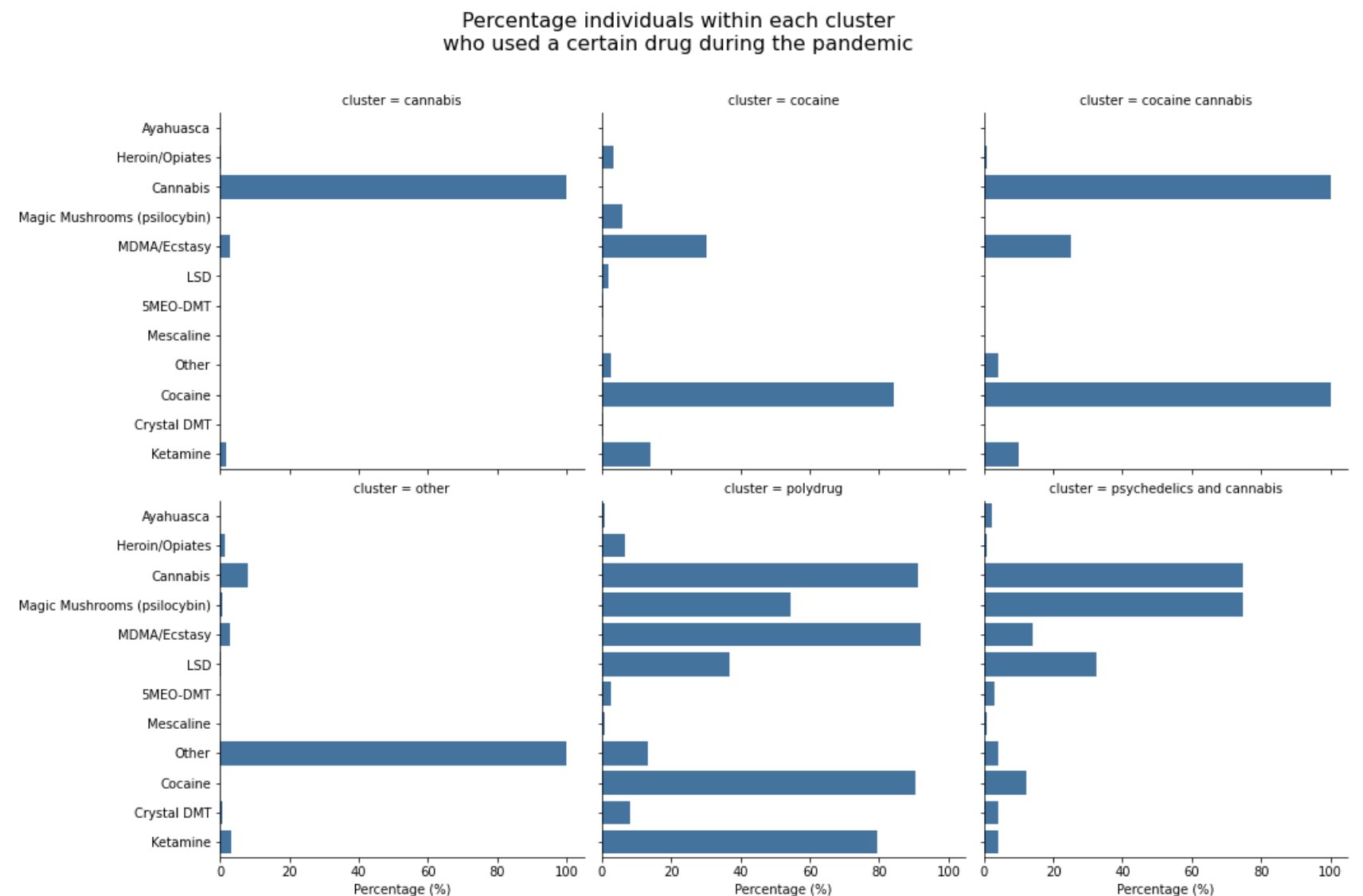


**Supplementary Figure S2. Data-driven clusters of drug use choices during the pandemic.** Percentages of individuals having consumed any of the drugs surveyed about within each cluster are represented.

| cluster | cannabis | cocaine | cocaine and cannabis | drug naive | historic user | polydrug | psychedelics and cannabis | stopped |
| --- | --- | --- | --- | --- | --- | --- | --- | --- |
| decade_10.0s | 6.560 | 2.610 | 5.260 | 2.750 | 0.380 | 11.850 | 7.020 | 3.530 |
| decade_20.0s | 21.940 | 34.310 | 31.100 | 10.340 | 9.010 | 49.630 | 36.400 | 22.820 |
| decade_30.0s | 23.880 | 30.720 | 24.880 | 13.070 | 19.300 | 25.190 | 21.490 | 21.530 |
| decade_40.0s | 17.930 | 21.570 | 26.320 | 14.900 | 24.100 | 9.630 | 18.420 | 20.820 |
| decade_50.0s | 16.990 | 8.820 | 10.050 | 24.740 | 25.000 | 3.700 | 10.530 | 17.290 |
| decade_60.0s | 10.300 | 1.960 | 2.390 | 23.800 | 17.960 | NaN | 6.140 | 11.760 |
| decade_70.0s | 2.340 | NaN | NaN | 9.320 | 4.120 | NaN | NaN | 2.240 |
| decade_>80s | 0.070 | NaN | NaN | 1.090 | 0.120 | NaN | NaN | NaN |
| Sex_Female | 34.390 | 30.390 | 23.330 | 47.070 | 35.990 | 22.060 | 32.460 | 32.940 |
| Sex_Male | 64.420 | 68.950 | 75.240 | 52.290 | 63.560 | 77.210 | 67.110 | 66.590 |
| Sex_Other | 1.190 | 0.650 | 1.430 | 0.640 | 0.450 | 0.740 | 0.440 | 0.470 |
| Education_Elementary_or_less | 2.010 | 1.310 | 4.310 | 1.700 | 1.400 | 0.740 | 0.880 | 1.290 |
| Education_High_school_diploma | 34.920 | 30.720 | 32.540 | 31.930 | 28.480 | 23.700 | 37.280 | 33.410 |
| Education_PhD | 4.480 | 0.980 | 1.910 | 6.030 | 5.900 | 4.440 | 4.390 | 6.120 |
| Education_University_degree | 58.600 | 66.990 | 61.240 | 60.340 | 64.220 | 71.110 | 57.460 | 59.180 |
| Ethnicity_American_Hispanic | 3.700 | 2.940 | 2.860 | 2.720 | 2.580 | 2.210 | 2.630 | 3.270 |
| Ethnicity_East_Asian | 2.640 | 5.560 | 1.430 | 2.710 | 3.080 | 5.150 | 2.630 | 3.500 |
| Ethnicity_Indian_South_Asian_South_East_Asian | 2.310 | 1.310 | 2.380 | 2.600 | 1.100 | 0.740 | 2.190 | 1.750 |
| Ethnicity_Mixed | 4.100 | 3.920 | 4.760 | 1.890 | 1.780 | 6.620 | 4.390 | 3.620 |
| Ethnicity_Other | 1.390 | NaN | 0.480 | 0.760 | 0.770 | NaN | 0.440 | 1.280 |
| Ethnicity_Unknown | 0.460 | 0.980 | NaN | 0.270 | 0.380 | NaN | 0.440 | 0.350 |
| Ethnicity_white | 85.400 | 85.290 | 88.100 | 89.040 | 90.310 | 85.290 | 87.280 | 86.230 |
| Occupation_Disabled_Sheltered_employment | 1.450 | 1.310 | 0.950 | 0.800 | 1.390 | 0.740 | 2.190 | 0.700 |
| Occupation_Homemaker | 2.240 | 0.330 | 0.950 | 2.550 | 2.110 | 0.740 | 0.440 | 1.400 |
| Occupation_Looking_for_work | 4.750 | 5.230 | 6.670 | 2.390 | 2.950 | 2.940 | 7.020 | 4.320 |
| Occupation_Retired | 8.780 | 0.980 | 1.900 | 28.770 | 17.320 | 2.210 | 3.510 | 10.970 |
| Occupation_Student | 13.730 | 10.780 | 12.860 | 6.090 | 3.330 | 30.150 | 21.050 | 10.850 |
| Occupation_Unknown | 1.780 | NaN | 2.380 | 1.480 | 0.470 | 0.740 | 0.440 | 1.280 |
| Occupation_Worker | 67.260 | 81.370 | 74.290 | 57.920 | 72.430 | 62.500 | 65.350 | 70.480 |
| Residence_Abroad | 13.730 | 4.580 | 3.330 | 5.450 | 6.610 | 6.620 | 16.230 | 5.950 |
| Residence_United Kingdom | 86.270 | 95.420 | 96.670 | 94.550 | 93.390 | 93.380 | 83.770 | 94.050 |
| Meditation_Almost never | 33.140 | 31.050 | 30.950 | 26.600 | 29.440 | 38.970 | 35.090 | 30.110 |
| Meditation_Daily | 2.970 | 1.630 | 2.380 | 3.870 | 3.610 | 5.150 | 7.020 | 2.800 |
| Meditation_Never | 48.120 | 62.090 | 59.050 | 57.000 | 54.790 | 43.380 | 34.210 | 52.390 |
| Meditation_Once or twice a week | 11.620 | 3.270 | 6.190 | 9.220 | 9.180 | 9.560 | 14.910 | 10.740 |
| Meditation_Several times a week | 4.160 | 1.960 | 1.430 | 3.310 | 2.980 | 2.940 | 8.770 | 3.970 |
| Reading_Almost never | 30.890 | 35.950 | 33.330 | 22.920 | 24.560 | 32.350 | 28.070 | 25.200 |
| Reading_Daily | 21.850 | 17.650 | 13.810 | 33.350 | 31.250 | 16.180 | 21.930 | 23.100 |
| Reading_Never | 6.670 | 8.500 | 9.050 | 4.700 | 4.510 | 12.500 | 2.630 | 6.070 |
| Reading_Once or twice a week | 25.350 | 26.140 | 29.050 | 22.950 | 23.560 | 20.590 | 27.190 | 25.550 |
| Reading_Several times a week | 15.250 | 11.760 | 14.760 | 16.080 | 16.120 | 18.380 | 20.180 | 20.070 |
| Exercise_Almost never | 21.190 | 20.260 | 19.050 | 17.200 | 19.360 | 18.380 | 13.600 | 21.820 |
| Exercise_Daily | 16.830 | 13.400 | 12.380 | 19.750 | 17.970 | 13.240 | 9.210 | 14.940 |
| Exercise_Never | 3.760 | 2.940 | 4.290 | 2.710 | 3.150 | 5.880 | 3.070 | 3.150 |
| Exercise_Once or twice a week | 29.310 | 31.700 | 29.520 | 30.850 | 30.030 | 33.090 | 39.910 | 32.440 |
| Exercise_Several times a week | 28.910 | 31.700 | 34.760 | 29.490 | 29.490 | 29.410 | 34.210 | 27.650 |

**Supplementary Table 1. Sociodemographic and lifestyle characteristics across timepoints.** Percentage of individuals within each cluster situated in each sociodemographic category are represented.

| **timepoint** | **cluster** | **N** |
| --- | --- | --- |
| **december2020** | drug naive | 7175 |
|  | historic user | 1559 |
|  | cannabis | 504 |
|  | stopped | 322 |
|  | cocaine | 90 |
|  | Psychedelics and cannabis | 70 |
|  | Cocaine and cannabis | 64 |
|  | polydrug | 33 |
| **january2022** | drug naive | 3573 |
|  | historic user | 2623 |
|  | cannabis | 619 |
|  | stopped | 317 |
|  | cocaine | 157 |
|  | Psychedelics and cannabis | 107 |
|  | Cocaine and cannabis | 99 |
|  | polydrug | 73 |
| **june2021** | drug naive | 4843 |
|  | historic user | 1556 |
|  | cannabis | 392 |
|  | stopped | 218 |
|  | cocaine | 59 |
|  | Psychedelics and cannabis | 51 |
|  | Cocaine and cannabis | 47 |
|  | polydrug | 30 |

**Supplementary table 2. Number of individuals with complete datasets from pre-pandemic to follow ups.**

| Cluster | Change | Count |
| --- | --- | --- |
| Cannabis | No change | 888 |
|  | I am using less | 329 |
|  | I am using more | 298 |
| Cocaine | I am using less | 142 |
|  | No change | 129 |
|  | I am using more | 35 |
| Cocaine and Cannabis | No change | 96 |
|  | I am using less | 65 |
|  | I am using more | 49 |
| Polydrug | No change | 52 |
|  | I am using more | 48 |
|  | I am using less | 36 |
| Psychedelics and Cannabis | No change | 131 |
|  | I am using less | 51 |
|  | I am using more | 46 |

**Supplementary Table 3. Number of individuals within each cluster and their self-reported changes in drug use since baseline.** Individuals were grouped based on their cluster, follow-up timepoint and self reported change.


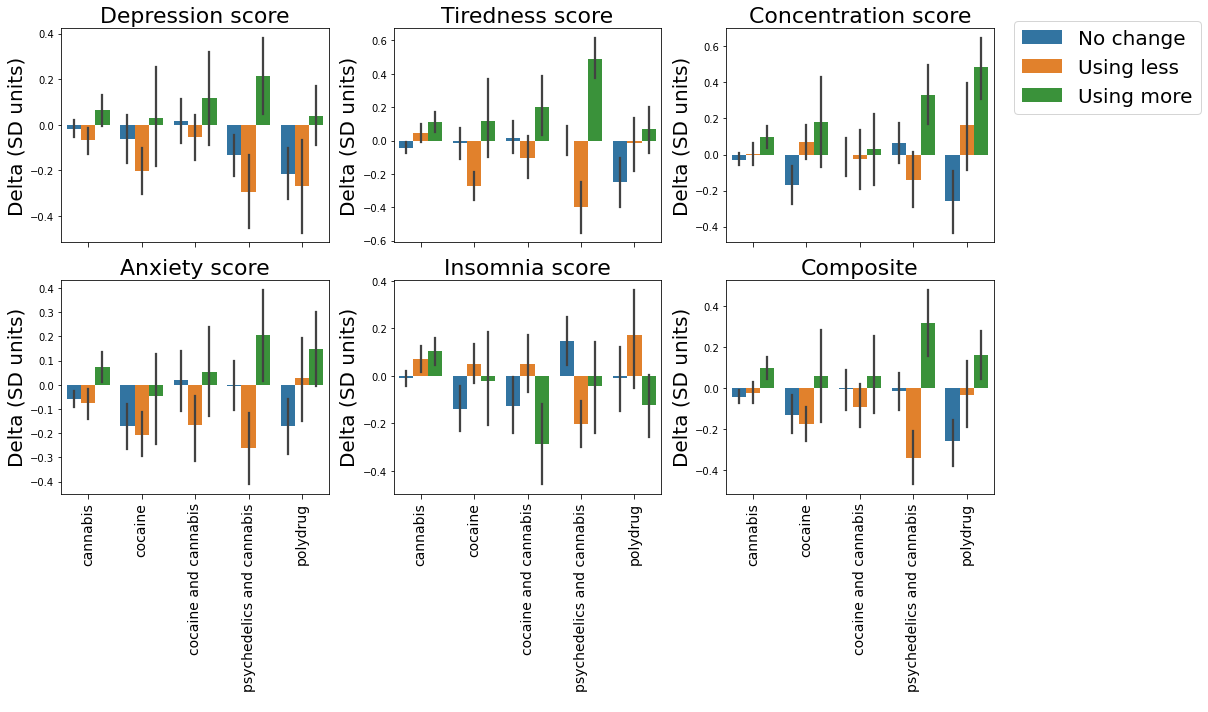


**Supplementary Figure S4. Changes in drug use deltas per drug cluster.** Changes in mental health are plotted across all timepoints for different clusters. Error bars are the standard error of the mean.

**Mixed effects models output**

Available as SupplementaryFile1.txt file

**Supplementary ANOVA analysis**

Available as SupplementaryFile2.txt file
